# Effect of Conjugated Linoleic Acid on Memory and Reflex Maturation in Rats Treated During Early Life

**DOI:** 10.3389/fnins.2019.00370

**Published:** 2019-04-24

**Authors:** Michelly Pires Queiroz, Martiniano da Silva Lima, Mayara Queiroga Barbosa, Marilia Ferreira Frazão Tavares de Melo, Camila Carolina de Menezes Santos Bertozzo, Maria Elieidy Gomes de Oliveira, Rui José Branquinho Bessa, Susana Paula Almeida Alves, Maria Izabel Amaral Souza, Rita de Cassia Ramos do Egypto Queiroga, Juliana Késsia Barbosa Soares

**Affiliations:** ^1^Program of Food Science and Technology, Federal University of Paraíba, João Pessoa, Brazil; ^2^Laboratory of Experimental Nutrition, Department of Nutrition, Federal University of Campina Grande, Campina Grande, Brazil; ^3^Centre for Interdisciplinary Research in Animal Health (CIISA), Faculty of Veterinary Medicine, University of Lisbon, Lisbon, Portugal; ^4^Program in Animal Science, School of Veterinary and Animal Science, Federal University of Goiás, Goiânia, Brazil; ^5^Laboratory of Bromatology, Department of Nutrition, Federal University of Paraíba, João Pessoa, Brazil

**Keywords:** conjugated linoleic acid, neurodevelopment, reflex maturation, memory, physical parameters, fatty acids, maternal nutrition

## Abstract

In the critical period of neurodevelopment (gestation and lactation), maternal consumption of essential fatty acids (FAs) can alter the offspring cognitive function permanently causing damage. Lipids can regulate neurotrophin and compose brain tissue. However, the effects of maternal consumption of a mixture of conjugated linoleic acid (CLA) on an offspring nervous system are not completely clear. We aimed to investigate the impacts of different CLA concentrations mixed into the maternal diet during early life on neonatal reflex maturation and cognitive functions of the offspring. Three groups were formed: control (CG): receiving a standard diet; CLA1: receiving a diet containing 1% of CLA, and CLA3: receiving a diet containing 3% of CLA, offered during gestation and lactation. After birth, the reflex responses of the offspring were observed from the 1st to the 21st day. After weaning, the animals’ anxiety and memory were assessed using open field (OF) and novel object recognition tests. Fatty acids in the breast milk and the offspring’s brain were also quantified. The data were analyzed using one-way ANOVA and the Kruskal–Wallis test. CLA1 presented accelerated palmar grasp disappearance versus CLA3 and negative-geotaxis versus CG; and the CLA3 presented increases for most reflexes (cliff-avoidance, vibrissa-placing, negative-geotaxis, and auditory-startle response), and decrease in reflexes palmar grasp and free-fall righting versus CG (*p* < 0.05). CLA3 group explored less of the OF in the second exposure. CLA1 and CLA3 presented an increased exploration ratio for new objects, which indicates memory improvement. The milk tested from CLA3 demonstrated an increase in polyunsaturated fatty acids (PUFAs), and a decrease in monounsaturated fatty acids. The amount of CLA in milk was greater in CLA1 and CLA3 and in the brain offspring both presented moderated amounts of CLA. Maternal treatment with the CLA mixture induced anticipated reflex maturation and improved memory in the offspring. Even though CLA was detected in the brains in only trace amounts, offspring’s brain PUFA and SFA levels were increased. Further studies aimed to delineate the effect of maternal CLA supplementation on offspring’s brain lipid metabolism and long-term neurologic outcome are needed to confirm these findings.

## Introduction

The central nervous system first appears in the human embryo at around the 3rd or 4th week after fertilization; development continues until roughly to 2 years of age. In rats, development occurs from the second week of pregnancy until the end of lactation. This phase is known as “the critical period of development” and any injury can cause permanent damage (Morgane et al., 1993; Hsieh and Brenna, 2009).

In this critical period of development, there is an increased need for polyunsaturated fatty acids (PUFAs) in brain; chiefly arachidonic acid (AA, 20:4n-6) and docosahexaenoic acid (DHA, 22:6n-3), which together comprise about 20% of the brain tissue (Valenzuela and Nieto, 2003). DHA provides better blood flow and optimizes the development and functions of the neuronal membrane (Valenzuela and Nieto, 2003; Balogun et al., 2014). Regular PUFA intake is important for neurotrophin regulation. Neurotrophins perform essential functions during the development of the fetal nervous system (Balogun et al., 2014). During lactation, breast milk replaces the placental function by carrying nutrients from the nursing mother to the neonate. The lipid fraction present in breast milk, in addition to its energy supply function, is also responsible for myelin sheath structuring.

Found in breast milk, linoleic (18:2n-6) and linolenic (18:3n-3) are PUFAs, and also essential fatty acids (FAs). Adequate intake of FAs is necessary for proper neurological and cognitive development in infants, and deficiencies during the brain development phase are associated with behavioral abnormalities (Hayat et al., 1999; Herrera, 2002; Gustavsson et al., 2010). Conjugated linoleic acids (CLAs) are a family of linoleic acid isomers presenting conjugated double bonds. CLAs are naturally produced by ruminant animals, found in their milk fats and muscle tissue, and in food products derived from them (Pariza et al., 2001; Banni, 2002). CLA isomers are commercially prepared by partial hydrogenation of linoleic acid (Banni, 2002). CLAs have been widely investigated due to their many beneficial health effects (Halade et al., 2010; Park et al., 2010; Furlan et al., 2013; Jelińska et al., 2014). It was found that CLAs cross the blood–brain barrier (Jelińska et al., 2014), inhibit angiogenesis in the mammalian brain (Sikorski et al., 2008), and *in vitro* were found to protect cortical cells against neurotoxic elements (Hunt et al., 2010). A maternal diet containing goat milk fat (as a source of CLAs), has also been found to affect cortical electrical activity (Soares et al., 2012) and anxiety in rats (Soares et al., 2013).

In the previous studies, goat milk was used as a source of CLA, but also of other lipids such as AA and DHA. Lipids of the n-3 series, present in goat’s milk, are already known for their beneficial effects on the nervous system when supplemented during pregnancy and lactation. Research has found improvement in the memory of pups whose mothers were supplemented during pregnancy with this lipid and with valproic acid. This medication is used indiscriminately in pregnancy and can cause adverse effects, such as fetal malformation and cognitive defects (Chalon et al., 1998). Improvement in brain development has also observed in mice receiving series-3 fatty acids (DHA, EPA, and AA) during lactation (Gaoa et al., 2016). However, studies analyzing the effects of CLA alone in these phases of life are still scarce.

It is well-known that dietary lipids, when offered during the initial phases of life, may alter reflexes, maturation (Santillan et al., 2010; Aquino et al., 2015), and behavior in animals (Soares et al., 2013). The hypothesis of this study is that maternal feed supplementation containing a mixture of CLA isomers during pregnancy and lactation positively influences reflex maturation (short term), and improves memory (long term) in rat offspring. This study aims to investigate the impact of supplementing the maternal diet with differing concentrations of a commercial CLA mixture on reflex ontogeny and memory in offspring.

## Materials and Methods

### Animals and Diets

Female Wistar rats (*n* = 12, four female per each group), acquired from the Federal University of Paraíba (UFPB), aged 90 days and weighing 230 ± 30 g were used to obtain pups (*n* = 36, only males were used). One female was maintained for each male during the mating period.

After confirmation of pregnancy, the mothers were housed in individual polypropylene maternity cages under standard conditions: temperature 22 ± 1°C, with a light-dark cycle (12 h; first light at 6:00 h), humidity of approximately 65%, and food and water *ad libitum*. During the first week of gestation, the rats received a commercial diet (Presence Purina^®^, São Paulo, Brazil), and an experimental diet was then offered starting from the second week of gestation and throughout lactation. During pregnancy and lactation, maternal feed intake and body weight were measured weekly. Three groups were formed: the control group (CG) receiving a standard diet without CLA (*n* = 11); the CLA1 group receiving an experimental diet containing 1% CLA (*n* = 13); and the CLA3 group receiving an experimental diet containing 3% CLA (*n* = 12) (Table 1); all diets were in accordance with the recommendations of the American Institute of Nutrition (AIN-93G) (Reeves et al., 1993). The CLA mix used was Clarinol^®^ powder (Stepan Lipid Nutrition, Maywood, NJ, United States); the composition is shown in Table 2.

**Table 1:** Composition of control and experimental diets.

Ingredient (g/kg)	Diets		
	Control	CLA1	CLA3
Corn starch	530	520	500
Casein	199.5	199.5	199.5
Sucrose	100	100	100
Soybean oil	70	70	70
CLA mix isomers	–	10	30
Fiber	50	50	50
Mineral mix	35	35	35
Vitamin mix	10	10	10
L-Cystine	3.0	3.0	3.0
Choline bitartrate	2.5	2.5	2.5
Gross energy (Joules)	16.568,6	16.777,8	17.196,2

**Table 2:** Fatty acid composition of the commercial CLA mix.

Fatty acid	Mean	Standard deviation
14:0	0.1	0.011
16:0	4.3	0.038
18:0	1.4	0.021
18:1c9	10.7	0.135
18:1c11	0.6	0.024
18:2n-6	1.0	0.027
20:0	0.2	0.014
CLA-c9t11	39.2	0.097
CLA-t10c12	38.3	0.071
CLA c/t and t/c isomers	2.5	0.128
CLA-trans,trans	1.5	0.042
22:0	0.2	0.010

The litters were standardized with six pups and some parameters were evaluated: Litter size, Number of males, Number of females, Birth weights. After weaning, at 21 days of age, the animals were separated in polypropylene cages, two animals per cage, where they received water and commercial feed *ad libitum*, containing 1.589,9 J of energy, 23 g of proteins (24.21%), 63 g of carbohydrates (66.31%), and 4 g of lipids (9.47%).

**FIGURE 1:**
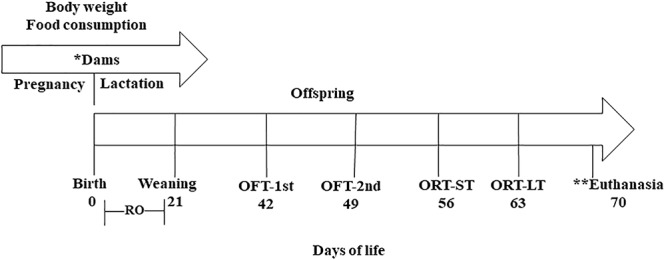
Experimental design. (^∗^) Treatment period where the mothers received: control diet (CG) (*n* = 11); diet containing 1% (*n* = 13); and diet containing 3% CLA (*n* = 12). (^∗∗^) Brain collection for fatty acid analysis. RO, reflex ontogeny; OFT-1st, open field test 1st exposure; OFT-2nd, open field test 2nd exposure; ORT-ST, object recognition test, short-term; ORT-LT, object recognition test, long-term.

The research followed an experimental protocol in accordance with the ethical recommendations of the National Institutes of Health (Bethesda, MD, United States), and was approved by the ethics research committee of the Federal University of Paraíba No. 0407/13 (Figure 1).

### Physical Maturation

The pups were weighed throughout lactation at 28, 49, and 70 days of life.

### Reflex Ontogeny in Newborn Pups

From the 1st day through the 21st postnatal day, the reflex responses were observed each day at 12:00 pm. Response consolidation was considered positive when a reflex reaction was repeated for three consecutive days (Smart and Dobbing, 1971), have established an experimental model for reflex maturation in rats, as presented in Table 3.

**Table 3:** Description of the reflex test.

Reflex	Stimulus	Response
Palmar grasp	Light percussion on the palm of the right foreleg	Quick bending of ankles
Righting	The rat is placed in supine position on a surface	Return to the prone position with all paws in 10s
Cliff-avoidance	The rat is placed on a flat and high surface (table), with legs toward the extremity	Moves to one side and walks in the opposite direction to the edge
Vibrissa-placing	The is was suspended by the tail and its vibrissae lightly touch the edge of a flat surface	Both front legs are placed on the table, performing march movements
Negative-geotaxis	The rat is placed at the center of an inclined ramp with head facing downwards	Body spin at an angle of 180°, positioning head upwards
Auditory-startle response	Intense and sudden sound stimulus	Retraction of anterior and posterior legs, with rapid and involuntary body immobilization
Free-fall righting	Held by the four legs, at a height of 30 cm, it is released in free fall on a synthetic foam bed	Position recovery during freefall on the surface supported on four paws

### Open Field Habituation Test

The open field habituation test is used to evaluate the animal to long-term habituation capacity on the open field device, consisting of a circular metallic arena (painted white) delimited by white walls with an open ceiling. The floor of the arena is divided into 17 fields (with lines painted black), 3 concentric circles (15, 34, and 55 cm in diameter, respectively) which are subdivided into a total of 16 segments and a central circle.

In rodents, habituation is analyzed by locomotor activity on the open field and is considered an indicator of non-associative learning (Leussis and Bolivar, 2006; Rachetti et al., 2013).

At 42 days of age, the animals were exposed to the open field in two phases, the second exposure occurred seven (7) days after the first. Four parameters were evaluated in the first and second expositions, each during 10 min (Rachetti et al., 2013; Gamberini et al., 2015; Speight et al., 2017).

• Duration of locomotion – Time spent by the animal moving in the open field.
• Number of crossings in open field – The ambulation was evaluated by the total of segments covered. It was counted when the animal inserted the four legs inside the segments.
• Number of entries into inner zone – Number of entries into inner zone – Quantified when the animal placed the four legs inside each inner zone of the open field.
• Duration in inner zone – Time spent by the animal in the inner zone of the open field.

All of the sessions were recorded with a video camera attached to the laboratory ceiling and the videos were analyzed afterward. The videos containing the data were analyzed randomly and by a single evaluator.

### Object Recognition Test

To evaluate declarative memory, when the animals reached 56 days of life, object recognition testing was applied on the previously used open field arena. Here, the animals underwent two open field exposures (the second exposure at 7 days after the first). The first test is associated with short-term memory, the second test relates to long-term memory (Rachetti et al., 2013).

The testing assesses the amount of time an animal spends in sniffing or touching an object with its nose and/or front legs. First, habituation is performed in the absence of any objects; animals may freely explore the arena for 3 min. Next, in the training session, the animals are placed in the arena when containing two different objects (A1 and A2) allowing free exploration for 10 min, for the animals to recognize and identify object A1 (a familiar object). The test session is held at 180 min after the training session to evaluate short-term memory, in which the animals are placed in the arena now containing two objects, A1 (the familiar object) and A3 (a new object), and they are allowed to freely explore for 5 min. After 7 days, another test is performed to assess long-term memory, in which the animals are placed in the arena to freely explore object A1 (the familiar object) together with object A4 (a new object). The videos were subsequently analyzed by a single evaluator on a random basis, that is, the evaluator was not aware of which group was being evaluated.

Before and after each test, the device and the objects were cleaned with 10% alcohol, and when exchanging animals, both the device and objects were cleaned with 10% alcohol and paper towels.

With completion of the test, the results obtained were analyzed using both the total time spent exploring the objects, and the novel object/(total familiar + novel object) ratio (Gustavsson et al., 2010).

### Profile of Brain and Milk Fatty Acids

The milk was collected at the end of lactation (21th day after weaning). Oxytocin (0,5 ml) was administered to facilitate lactation. For collection, the animals were anesthetized with ketamine hydrochloride and xilasin (1 ml/kg body weight). The milk was removed by hand (squeezing the rat’s breast) and placed in encoded Eppendorf tubes, 3 per group, where 2 CG Eppendorf tubes and 1 Eppendorf tube for CLA3 were lost during the journey, leaving 1 sample of CG, 3 samples of CLA1 and 2 of CLA3. After collection, the rats were sacrificed by cervical detachment.

At the end of the experiment, at 70 days of age and after a 6-h fast, the offspring were anesthetized and sacrificed. After euthanasia, the brain was removed using a scalpel and pliers, and then lyophilized.

Milk and brain samples were sent to the Faculty of Veterinary Medicine at the University of Lisbon where the FA analyses were conducted. Fatty acid methyl esters (FAMEs) from the freeze-dried milk fat samples were prepared by direct trans-esterification using potassium hydroxide (2M) in methanol, in accordance with (Rego et al., 2009) and FAMEs and dimethyl acetal (DMA) from the brain samples were prepared by reaction with HCl 1.25 M in methanol for 20 h at 50°C. Fatty acid methyl esters and DMA were analyzed by gas chromatography with flame ionization detection using a Shimadzu GC 2010-Plus (Shimadzu, Kyoto, Japan) equipped with a SP-2560 (100 m × 0.25 mm, 0.20 μm film thickness, Supelco, Bellefonte, PA, United States) capillary column. The chromatographic conditions were as follows: injector and detector temperatures were set at 250 and 280°C, respectively; helium was used as the carrier gas at 1 mL/min constant flow; the initial oven temperature of 50°C was held for 1 min, increased at 50°C/min to 150°C and held for 20 min; then increased at 1°C/min to 190°C; and finally increased at 2°C/min to 220°C and held for 40 min. Identification of FAME and DMA were achieved using electron impact mass spectrometry using a Shimadzu GC–MS QP2010 Plus (Shimadzu) and published chromatograms (Alves et al., 2013). The chromatographic column and the GC conditions were similar to the GC-FID analyses. Additional mass spectrometer conditions were as follows: ion source temperature, 200°C; interface temperature, 240°C; and emission voltage, 70 eV. The fatty acids and DMA inside the incubation tubes were expressed as milligrams per flask, and determined using the internal standard, assuming a direct proportionality between GC-FID peak area and FA weight.

### Statistical Analysis

Intergroup differences for reflex maturation were analyzed using one-way Kruskal–Wallis testing, followed by Dunn testing on Sigma Stat software (San Jose, CA, United States). Milk sample FA profile data were presented as means and as standard deviations when more than one sample per treatment was available. Brain tissue FA profile data were analyzed using PROC MIXED, SAS 9.4 (SAS Inst., Cary, NC, United States) using a model that considered the treatment as a single fixed effect, and allowed for variance heterogeneity between treatments. When significant (*p* < 0.05) treatment effects were detected the least square means were compared using the Tukey procedure.

## Results

### Maternal Feed Intake and Pup Weights

There were no significant differences in maternal food intake and body weight among the different groups.

**FIGURE 2:**
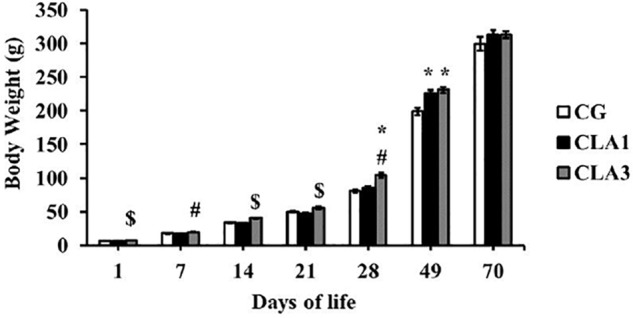
Body weight of rats during lactation to the beginning of adulthood, treated with diet containing 1% CLA (CLA1) or 3% CLA (CLA3). Values are expressed as means and standard error (one way ANOVA, Holm–Sidak); ^∗^ versus CG ^#^ versus CLA1 group; ^$^ versus all group.

Body weight analyses showed that the CLA3 group of pups presented higher body weights than the other two groups at 1, 14, and 21 days of age. However on day 7, the CLA3 group body weight was significantly higher only as compared to the CLA1 group, and not to the CG (*p* < 0.05). When assessing body weight after lactation, when animals were 28 days old, CLA3 had a higher body weight when compared to CLA1 and CG. At 49 days of age, the animals of the CLA1 and CLA3 groups presented higher body weights versus CG (*p* < 0.05). At the end of the experiment no significant statistical differences were observed between the groups (Figure 2).

### Birth Data

Table 4 shows the parameters evaluated after birth. The CLA1 and CLA3 groups presented larger litters as compared to CG (*p* < 0.05). For the other parameters, there were no significant statistical differences.

**Table 4:** Evaluation of parameters after birth.

Birth data	Control	CLA1	CLA3
Litter size^∗^	8.0 ± 3.6	13.0 ± 0.9^∗^	13.0 ± 0.5^∗^
Number of males	4.0 ± 1.0	7.0 ± 2.6	7.0 ± 0.5
Number of females	4.0 ± 2.7	6.0 ± 1.9	6.0 ± 1.1
Birth weights (g)	6.0 ± 0.6	6.0 ± 0.2	7.0 ± 0.9

^∗^*Three litters per each group*.

### Reflex Ontogeny in Newborn Pups

From evaluation of reflex maturation as measured in this study (Table 5), we observed that palmar grasp disappearance in the CLA1 and CLA3 groups was delayed as compared to the CG; and the CLA3 group was delayed as compared to the CLA1 group (*p* < 0.05). When maturation of cliff avoidance, vibrissa placing, negative geotaxis, and auditory startle were investigated, the CLA3 group presented acceleration as compared to the control group (CG) (*p* < 0.05). The CLA3 animals also presented increased righting and vibrissa placing in relation to the CLA1 group (*p* < 0.05). CLA1 showed acceleration of palmar grasp versus CLA3 and negative-geotaxis versus CG (*p* < 0.05). In summary, the CLA3 presented acceleration of four reflexes and CLA1 three reflexes of the seven evaluated.

**Table 5:** Reflex maturation of rats which mothers were treated during gestation and lactation with standard diet containing soybean oil and other two groups with experimental diets, one containing 1% CLA (CLA1) and the other with 3% CLA (CLA3).

Reflex maturation	Diets		
	Control (*n* = 11)	CLA1 (*n* = 16)	CLA3 (*n* = 12)
Palmar grasp^a^	3 (3–6)	3.5 (2–10)^∗^	5 (2–10)^∗^#
Righting^b^	3 (1–6)	3 (2–6)	2 (1–4)#
Cliff-avoidance^b^	10 (6–13)	7.5 (3–13)	6 (5–7)^∗^
Vibrissa-placing^b^	11 (8–12)	10 (4–13)	6 (3–10)^∗^#
Negative-geotaxis	17 (8–18)	13 (11–17)^∗^	13 (8–16)^∗^
Auditory-startle response^b^	14 (12–17)	13 (12–15)	12 (12–13)^∗^
Free-fall righting^b^	11 (8–15)	12 (9–14)	13 (10–16)^∗^

*Values expressed as median (minimum–maximum); (one-way ANOVA, Kruskal–Wallis); ^∗^ versus Control group; ^#^ versus CLA1; ^*a*^desappearance; ^*b*^appearance*.

### Open Field Habituation

**FIGURE 3:**
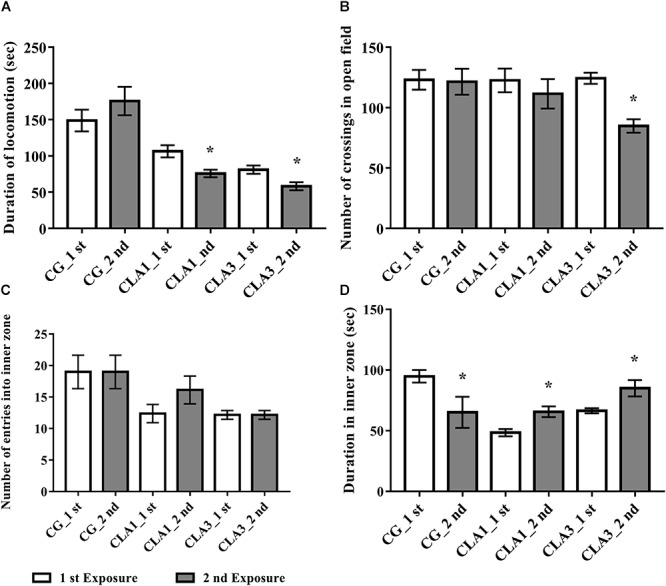
Habituation test with newborn rats treated with standard diet (CG), with 1% CLA (CLA1), or 3% CLA (CLA3) during pregnancy and lactation (maternal diet). Values are expressed as means and standard deviation (one way ANOVA, Holm–Sidak); 1st exposure to 42 days of life and 2nd exposure to 49 days of life; CG (*n* = 11), CLA1 (*n* = 13), CLA3 (*n* = 12); ^∗^ versus first exposure in the same group. **(A)** Duration of locomotion: time spent by the animal moving in the open field. **(B)** Number of crossings in open field: the ambulation was evaluated by the total of segments covered. It was counted when the animal inserted the four legs inside the segments. **(C)** Number of entries into inner zone: quantified when the animal placed the four legs inside each inner zone of the open field. **(D)** Duration in inner zone – time spent by the animal in the inner zone of the open field.

In Figure 3A, the CLA1 and CLA3 groups spent less time ambulating in the second exposure to the open field when compared to the first exposure (*p* < 0.05). In Figure 3B, it is possible to observe a smaller number of crossings in the fields of the open field in CLA3 in the second exposure (*p* < 0.05). The other groups did not present a statistically significant difference (*p* > 0.05). When the time spent in the internal zone was evaluated, CG spent less time in the internal zone when compared to the first and second exposures. CLA1 and CLA3 spent a longer time in the internal zone when comparing the two exposures (*p* < 0.05) (Figure 3D). The number of entry in the internal zone did not differ between the groups (Figure 3C).

### Object Recognition

**FIGURE 4:**
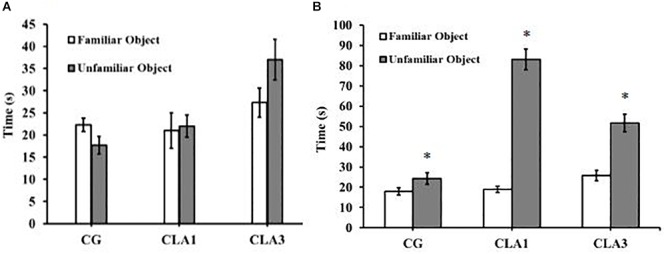
Object recognition test in rats treated with 1% (CLA1) or 3% CLA (CLA3) during pregnancy and lactation (maternal diet). CG: Control group without CLA in the diet. Values are expressed as means and standard deviation (one way ANOVA, Holm–Sidak). **(A)** Short-term test using familiar object (A1) and unfamiliar object (A3). **(B)** Long-term test using familiar object (A1) and unfamiliar object (A4); CG (*n* = 11), CLA1 (*n* = 13), CLA3 (*n* = 12); ^∗^ versus familiar object in the same group.

**FIGURE 5:**
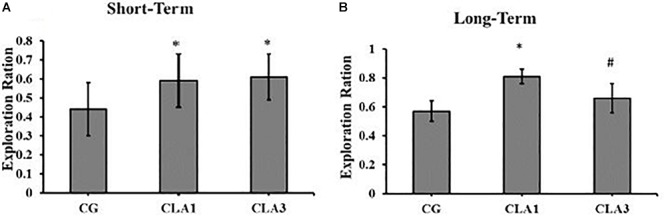
Exploration Ratio for object recognition test in rats treated during pregnancy and lactation (maternal diet) with 1% (CLA1) or 3% CLA (CLA3). CG: Control group without CLA in the maternal diet. Values are expressed as means and standard deviation (one way ANOVA, Holm–Sidak). **(A)** Short-term test using familiar object (A1) and unfamiliar object (A3). **(B)** Long-term test using familiar object (A1) and unfamiliar object (A4); CG (*n* = 11), CLA1 (*n* = 13), CLA3 (*n* = 12); ^∗^ versus CG; ^#^ versus CLA1 group.

For short-term memory testing, there were no significant differences between the groups (Figure 4A). In the long-term memory test, all groups (CG, CLA1, and CLA3) explored unfamiliar objects more than familiar objects (Figure 4B) (*p* = 0.0373), the CLA1 group presented an increased long-term exploration ratio as compared to the CG and the CLA3 group (Figure 5B). In the short-term CLA1 and CLA3 presented higher exploration ratio compared with CG (*p* < 0.05) (Figure 5A).

### Fatty Acid Profiles in Milk

The means and standard deviations of FAs obtained from the available milk samples are presented in Table 6. The FA milk profiles from the animals (three differing diets) was in general similar, although a decrease in *cis*-monounsaturated fatty acid (MUFA), and an increase of total PUFA with CLA3 treatment as compared to the other treatments is suggested. The increase in total PUFA was due to the proportional increase of CLA in the milk presented by maternal (lactating) rats fed CLA supplemented diets. In fact, the control milk samples contained only trace amounts of the 18:2c9,t11 isomer, and presented no 18:2t10c12, whereas the CLA1 and CLA3 group milk samples respectively contained about 1 and 3% of total FA for each CLA isomer.

**Table 6:** Fatty acid composition (means ± standard deviation expressed as g/kg of total fatty acids) of breast milk of dams fed control, CLA1 and CLA2 diets.

Fatty acids	Control	CLA1	CLA3
n^1^	1	3	2
8:0	29.5	37.9 ± 11.1	52.5 ± 3.07
10:0	92.7	104 ± 42.9	148 ± 15.9
12:0	61.9	56.0 ± 27.3	80.1 ± 14.2
14:0	54.3	41.3 ± 15.7	49.9 ± 10.4
15:0	1.05	1.24 ± 0.18	1.12 ± 0.29
16:0	219	201 ± 24.0	159 ± 3.3
17:0	0.98	1.16 ± 0.20	1.03 ± 0.19
18:0	31.0	36.4 ± 4.44	31.5 ± 1.77
20:0	0.72	0.80 ± 0.07	0.95 ± 0.04
22:0	0.20	0.38 ± 0.04	0.28 ± 0.02
23:0	0.23	0.29 ± 0.05	0.31 ± 0.10
24:0	0.36	0.40 ± 0.04	0.51 ± 0.06
SFA^2^	492	481 ± 68.6	524 ± 44.6
14:1*c*9	1.00	0.74 ± 0.29	0.34 ± 0.12
16:1*c*7	3.25	3.05 ± 0.90	1.99 ± 0.39
16:1*c*9	36.4	26.6 ± 9.27	12.0 ± 1.50
17:1*c*9	1.16	1.09 ± 0.29	0.68 ± 0.14
18:1*t*6/*t*7/*t*8	0.17	0.29 ± 0.02	0.25 ± 0.02
18:1*t*9	0.22	0.27 ± 0.08	0.23 ± 0.01
18:1*t*10	0.17	0.35 ± 0.09	0.56 ± 0.16
18:1*t*11	0.53	0.68 ± 0.01	1.01 ± 0.44
18:1*t*12	0.24	0.41 ± 0.06	0.34 ± 0.01
18:1*c*9	230	222 ± 48.1	159 ± 19.5
18:1*c*11	17.6	18.1 ± 4.40	11.7 ± 1.89
18:1*c*13	0.46	0.52 ± 0.03	0.42 ± 0.18
24:1*c*15	0.10	0.23 ± 0.10	0.11 ± 0.004
*cis*-MUFA^3^	290	273 ± 61.3	186 ± 20.6
*trans*-MUFA^4^	1.34	2.27 ± 019	3.05 ± 0.61
18:2n-6	187	193 ± 15.3	187 ± 18.1
18:3n-6	1.83	1.41 ± 0.76	0.94 ± 0.08
20:2n-6	2.67	3.43 ± 0.47	3.14 ± 0.30
20:3n-6	1.59	1.63 ± 0.58	1.06 ± 0.05
20:4n-6	5.35	6.52 ± 1.70	6.26 ± 0.64
n-6 PUFA^5^	199	206 ± 18.1	198 ± 19.0
18:3n-3	13.0	14.7 ± 2.47	16.4 ± 0.78
20:3n-3	0.43	0.47 ± 0.06	0.50 ± 0.03
20:5n-3	0.38	0.42 ± 0.08	0.49 ± 0.01
22:5n-3	0.63	0.70 ± 0.09	0.66 ± 0.09
22:6n-3	0.78	1.06 ± 0.43	1.24 ± 0.01
n-3 PUFA^6^	15.2	17.3 ± 2.77	19.3 ± 0.69
18:2*c*9*t*11	0.42	10.6 ± 1.84	35.0 ± 1.86
18:2*t*10*c*12	0.00	7.8 ± 1.85	29.6 ± 1.03
18:2*c*9*c*11	0.19	0.53 ± 0.09	0.91 ± 0.13
Other CLA	0.50	1.17 ± 0.06	2.22 ± 0.44
Total CLA	1.11	20.1 ± 3.73	67.8 ± 3.47
Total PUFA	216	244 ± 18.2	286 ± 23.4

^1^*Number of samples; ^*2*^Sum of saturated fatty acids; ^*3*^Sum of *cis* monounsaturated fatty acids; ^*4*^Sum of *trans* monounsaturated fatty acids; ^*5*^Sum of n-6 polyunsaturated fatty acids; ^*6*^Sum of n-3 polyunsaturated fatty acids*.

### Fatty Acids and Dimethyl Acetal in the Brain

The fatty acid and DMA profiles in the brain tissue of the pups are presented in Table 7. The treatments significantly affected most of the FA and DMA profiles, although they did not follow the expected response patterns. In fact, most of the FA and DMA profiles differed between the controls and the CLA1 pups, whereas the CLA3 pups were either similar to the controls (14:0, 23:0, 16:1c7, 18:1c9, 18:1c11, 20:1c11, 24:1c15, 18:2n-6, 20:2n-6, 20:3n-6, *cis-*MUFA, n-6 PUFA, total PUFA, DMA-17:0, DMA-18:1c9, DMA-18:1c11, and total DMA), or between the controls and the CLA1 pups (16:0, 20:0, 24:0, 16:1c9, 20:1, 22:1c13, 24:1, 20:4n-6, 22:4n-6, 22:5n-6, 22:6n-3, SFA, n-3 PUFA, and DMA-18:0). Thus, the brains of the CLA1 pups presented higher (*p* < 0.05) proportions of SFA and PUFA, but lower (*p* < 0.05) proportions of MUFA and total DMA than the controls. The CLA isomers were not detected in the brains of the CG pups, and were present only as trace amounts in the CLA1 and CLA3 pups.

**Table 7:** Fatty acids (FAs) and dimethyl acetals (DMAs) composition (least square means ± standard error, expressed as g/kg of total FA + DMA) cerebral tissue of offspring from dams fed control, CLA1 and CLA3 diets.

	Control	CLA1	CLA3	*P*-value
n^1^	6	4	4	
FA				
14:0	1.13 ± 0.07	3.54 ± 0.21*	1.77 ± 0.43#	<0.001
15:0	0.38 ± 0.03	0.63 ± 0.06*	0.52 ± 0.02*	0.003
16:0	179 ± 0.8	216 ± 4.0*	197 ± 10.8	<0.001
17:0	1.52 ± 0.04	1.40 ± 0.08	1.65 ± 0.17	0.289
18:0	207 ± 0.9	212 ± 1.92*	211 ± 1.5	0.025
20:0	5.86 0.08	4.25 0.35*	5.02 0.54	0.003
22:0	5.87 ± 0.12	4.93 ± 0.41	5.08 ± 0.52	0.075
23:0	1.60 ± 0.07	0.45 ± 0.09*	1.23 ± 0.33#	<0.001
24:0	12.3 ± 0.16	8.39 ± 0.87*	9.75 ± 1.41	0.002
SFA^2^	414 ± 0.6	452 ± 3.7*	433 ± 9.7	<0.001
16:1*c*7	1.21 ± 0.06	3.12 ± 0.11*	1.82 ± 0.44	<0.001
16:1*c*9	3.12 ± 0.18	4.06 ± 0.01*	3.55 ± 0.28	0.001
17:1*c*9	0.36 ± 0.04	0.27 ± 0.05	0.44 ± 0.08	0.209
18:1*trans*	2.18 ± 0.16	2.75 ± 0.16	2.23 ± 0.29	0.066
18:1*c*9	154 ± 0.6	122 ± 3.1*	141 ± 7.9#	<0.001
18:1*c*11	29.7 ± 0.17	24.9 ± 0.61*	28.7 ± 0.93#	<0.001
20:1*c*11^3^	12.9 ± 0.21	5.46 ± 0.49*	9.79 ± 1.87#	<0.001
20:1^4^	4.03 ± 0.05	1.77 ± 0.19*	3.08 ± 0.58	<0.001
22:1c13	1.85 ± 0.05	0.95 ± 0.08*	1.46 ± 0.28	0.012
24:1*c*15	18.1 ± 0.46	7.41 ± 0.84*	14.3 ± 2.96#	<0.001
24:1^4^	1.92 ± 0.05	0.94 ± 0.09*	1.58 ± 0.32	<0.001
*cis*-MUFA^5^	230 ± 0.8	173 ± 5.4*	208 ± 14.1#	<0.001
18:2n-6	6.12 ± 0.06	12.1 ± 0.32*	7.00 ± 1.25#	<0.001
20:2n-6	1.32 ± 0.07	2.58 ± 0.09*	1.50 ± 0.20#	<0.001
20:3n-6	3.51 ± 0.08	5.97 ± 0.34*	3.65 ± 0.26#	<0.001
20:4n-6	98 ± 0.65	115 ± 2.12*	104 ± 4.47	<0.001
22:4n-6	29.1 ± 0.27	30.9 ± 0.52	29.7 ± 0.55	0.029
22:5n-6	7.05 ± 0.21	9.13 ± 0.27*	7.68 ± 0.87	<0.001
n-6 PUFA^6^	145 ± 1.0	176 ± 2.7*	154 ± 7.4#	<0.001
22:5n-3	2.33 ± 0.04	3.19 ± 0.18*	2.14 ± 0.05*#	<0.001
22:6n-3	116 ± 0.9	123 ± 1.9*	120 ± 3.2	0.017
n-3 PUFA^7^	118 ± 0.9	127 ± 1.8	122 ± 3.1	0.030
20:2^4^	1.09 ± 0.05	0.79 ± 0.06*	0.86 ± 0.03*	0.005
20:3n-9	0.82 ± 0.06	0.69 ± 0.02	0.66 ± 0.03	0.101
18:2*c*9*t*11	nd	0.08 ± 0.06	0.24 ± 0.08	0.154
18:2*t*10*c*12	nd	0.05 ± 0.04	0.15 ± 0.05	0.171
Total CLA	nd	0.13 ± 0.09	0.39 ± 0.13	0.159
Total PUFA	266 ± 0.9	304 ± 3.9*	277 ± 10.3#	<0.001
DMA				
16:0	20.6 ± 0.35	23.8 ± 0.27*	22.1 ± 0.27*#	<0.001
17:0	0.85 ± 0.05	0.45 ± 0.06*	0.84 ± 0.16#	<0.001
18:0	42.3 ± 0.36	33.4 ± 0.62*	37.7 ± 2.69	<0.001
18:1*c*9	15.4 ± 0.15	8.1 ± 0.57*	12.4 ± 1.95#	<0.001
18:1*c*11	11.5 ± 0.18	5.01 ± 0.29*	9.14 ± 1.72#	<0.001
Total DMA	90.6 ± 0.66	70.8 ± 1.44*	82.3 ± 6.20#	<0.001

^1^*number of samples; ^*2*^Sum of saturated fatty acids; ^*3*^Coelutes with minor amounts of 18:3n-3; ^*4*^Double bond position not determined; ^*5*^Sum of *cis*-monounsaturated fatty acids; ^*6*^Sum of n-6 polyunsaturated fatty acids; ^*7*^Sum of n-3 polyunsaturated fatty acids; ^∗^ versus CG ^#^ versus CLA1 group*.

## Discussion

This study evaluated the developmental effects of maternal supplementation with differing CLA concentrations (1 and 3%) administered during gestation and lactation in their offspring. The data presented the significant effects of CLA on physical growth (with increases in body weight), and on neurodevelopment (acceleration in reflex maturation and memory improvement) in the newborn rats.

Neurodevelopment occurs in the perinatal period, and lipids are increasingly recognized as playing an important role in neuronal function in the brain (Morgane et al., 1993; Salvati et al., 2000). During this phase, essential fatty acids of the n-3 and n-6 families together constitute a lipid substrate that is required for adequate formation of nerve cell membranes. The lipids are also involved in cell signaling and regulate synaptic throughput (Muller et al., 2015). The effects of n-3 in the maternal diet on brain development of the offspring are already known (Rathod et al., 2014; Mucci et al., 2015), However, the effect of other fatty acids like CLA have not yet been investigated. In this study, a maternal diet that included CLA, induced improvements in memory, and accelerated neurodevelopment in the offspring.

Maternal diets containing different n-6/n-3 ratios in rats have been associated with reflex ontogeny and physical growth modifications in the offspring (Santillan et al., 2010; Aquino et al., 2015). Such results demonstrate early life vulnerability of developing nervous systems to an inadequate balance of essential fatty acids. Treatment of Wistar rats during lactation with goat milk (fat containing CLA) has been shown to result in cliff avoidance delays, but there were anticipations in free-fall righting (Soares et al., 2013). Another study (Santillan et al., 2010) found a delay for negative geotaxis in animals consuming soybean oil, yet an acceleration for cliff avoidance in animals treated with sunflower oil. The cerebellum presents peak development during lactation, and reflex maturity is directly related to the continuous differentiation and maturation of cerebellar neurons (Allam and Albo-Eleneen, 2012), which involve the visual and postural systems (Boyle, 2001).

In the present study, 1% CLA induced acceleration for palmar gasp and negative geotaxis, however when the dose was tripled, cliff-avoidance, vibrissa-placing, negative-geotaxis, auditory-startle response, were accelerated. The group treated with CLA3% obtained a better response in four parameters of the seven evaluated for reflex. On the other hand, the CLA1 group presented two accelerated reflexes. When analyzing the two groups it can be stated that the effects were moderate. It is unclear what mechanisms the body uses to transform CLA into PUFAs, what is known is that no large doses of omega-3 lipids are required for a better response in the nervous system. In addition, high doses of this lipid may cause depletion of omega-6 lipids and the consequent imbalance between n-3 and n-6 (Zanarini and Frankenburg, 2003; Salvati et al., 2006). These findings suggest that CLA positively affects neurodevelopment, anticipating reflex maturation in the offspring.

Studies show that CLA can pass through the blood–brain barrier (Fa et al., 2005; Soares et al., 2012), reaching the brain, where it performs beneficial functions. However in our study, CLA was found in the brain only in trace amounts. In the milk, in the CLA1 and CLA3 groups, apparently the quantities of CLA were higher, but this cannot be stated with accuracy since statistical analysis was not performed, since some of the samples were lost during transit. This may be considered a limitation of our study.

Thus, we believe that in the present study the effects of this fatty acid occurred indirectly. Study analyzed aspects of maternal metabolism on milk composition show that the transfer of fatty acids to the mother’s milk may vary depending on their quality as consumed in the maternal diet (Demmelmair and Koletzko, 2018). The authors observed that alpha-linolenic acid offered in the diet contributes more than that of the normal maternal reserve to its final quantity as found in the milk. However, linoleic acid in breast milk is derived more from the liver than from the diet itself (Demmelmair and Koletzko, 2018). The literature reports on metabolism and omega 3 and 6 in breast milk (Demmelmair and Koletzko, 2018). However, little is known about the influence of maternal consumption of CLA on the composition of breast milk.

In rats fed a diet rich in *trans*-FAs, offered during early life and after weaning, spatial memory was modified (Souza et al., 2012), and a maternal diet containing high levels of lard and saturated fats was found to induce damages to the memory and learning ability of the offspring (Yu et al., 2010). However, both studies cited, evaluated memory using the Morris water maze test. In our study, new object recognition test was used to assess working memory. Both groups treated with CLA (CLA1 and CLA3) demonstrated increased time of exploration of unfamiliar (novel) objects, indicating a better working memory. This findings differ from the results reported by Yu et al. (2010), who showed that offsprings of mice treated with saturated fats performed worse in Morris water maze test. We should point out that we did different memory test and CLA isomers are unsaturated fat.

In early stage of life in the rat, neurodevelopment happens very fast, as demonstrated when was measured the reflex maturation in the present study. The reflex maturation occurred from birth to 18 days of life. In addition, maternal supplementation with CLA altered the deposition of fatty acids in the brain as demonstrated in Table 7. Unfortunately, we evaluated the composition of fatty acids in this tissue only at the end of the experiment (70 days of life), which made it impossible to evaluate the transition of fatty acids in this brain tissue and to relate to the transitional period that led to the consolidation of the memory by the animals. However, the exploration index by CLA groups were always higher than CG (short and long time). Thus, these data confirm how neurodevelopment occurs fast in the nervous system, even during lactation with the evaluation of reflex maturation and in the postnatal period with evaluated of memory by the object recognition test.

The experimental diets significantly affected most of the fatty acids and DMA, although they did not follow the expected response patterns. The CLA1 group presented higher amounts of PUFAs as compared to the CG, and the CLA3 group’s PUFA levels were intermediates between those of the CLA1 and CG groups. Among the polyunsaturated fats found in the brain for the CLA1 group we detected AA and DHA. In the brain, DHA (22:6n-3) and AA (20:4n-6) have been correlated with better spatial memory performance (Fernandes et al., 2011; Harauma et al., 2017).

As was seen in the results, CLA is present in breast milk, and it may be affirmed that the pups received CLA by breastfeeding. However, in the brain, the lipid was found only in small amounts. Yet maternal consumption of CLA induced memory improvement in their offspring. Although CLA was not deposited in large amounts in the brain, its consumption significantly increased n-3 and n-6 fatty acids in the rat brain. Among these fatty acids is AA, an n-6 fatty acid that plays an important role in brain functions, including neuronal signal transmission and long-term potentiation. In addition, AA preserves hippocampal neuron membrane plasticity, protects the brain against oxidative stress, improves memory, and helps in the synthesis of new proteins in brain tissues (Hadley et al., 2016).

AA can be synthesized from substrates such as CLA and LA (Banni, 2002). These fatty acids likely share the same enzymes (desaturases and elongases). Thus, increased CLA ingestion may impede metabolism of linoleic acid, and consequently pro-inflammatory metabolites (Białek et al., 2015).

The principally known metabolites of CLA belong to conjugated diene (CD) structured compounds, such as conjugated octadecetrienoic acid (CD18: 3), conjugated eicosatrienoic acid (CD 20: 3), and conjugated eicosatetraenoic acid (CD 20: 4) which are synthesized in the desaturase and elongase pathways (Banni, 2002). However, CLA metabolism may interfere with the formation of eicosanoids, and CLA hydroxylation and its metabolites conjugated in LOX pathways, or cytochrome P450 may form eicosanoid-like molecules that compete with regular eicosanoids (Banni, 2002), thus potentially exerting anti-inflammatory action in many, including brain tissue (Shen et al., 2018). Further studies would be needed to elucidate the mechanism by which CLA can induce increased DHA.

Polyunsaturated fatty acids are essential for brain development and memory because they modulate synaptic plasticity, and thus improve learning ability. In human infants (our study investigates this same stage of life), accumulation of these fatty acids occurs during gestation and lactation, through the placenta and breast milk (Banni et al., 1996).

Another memory index used was the open field habituation test, and lower locomotor activity in a repeated exposure indicates good recognition (Rachetti et al., 2013). In the present study CLA1 and CLA3 presented less locomotion and spent longer time in the internal area in the second exposure into the open field. This behavior indicates that the animals moved less because they remembered the place and lost interest in exploration. The time spent in the central area confirms that this decrease in locomotion in the second moment did not occur due to the behavior like anxiogenic of the animals.

A diet enriched in n-3 PUFA has induced an increase in exploratory activity for young rats, which was not observed in the mature or older rats tested (Das, 2003). However, a maternal CLA enriched diet reduced ambulatory activity, as demonstrated in the present study. Thus, it is demonstrated for the first time that CLA is able to affect these learning parameters. With greater CLA levels in the diet, levels of CLA isomers (18:2 *cis*-9, *trans*-11 and 18:2 *trans*-10, *cis*-12) increase in the milk. Yet in the brain, these CLA isomers were present only in trace amounts; and although proportionally higher in the CLA3 group as compared to the CLA1 group, the difference was not significant.

Maternal dietary lipids may also affect body weight in offspring. In this study, the body weight of pups whose mothers received 3% CLA was higher throughout and after lactation. A similar result was observed when during gestation and lactation (Soares et al., 2012) rats received a diet containing goat milk (which is a source of CLA). A different result was observed when lactating rats received 1.35% CLA (Hayashi et al., 2007). Maternal treatment during pregnancy and lactation with 1.47% CLA resulted in pups presenting decreased body weights (Ringseis et al., 2004). The authors attribute this effect to lipid reduction in the breast milk; and yet such a reduction was not induced in the present study. The data demonstrate that maternal dietary lipids may differently affect the physical parameters of their offspring. Evaluating the body weight and feed intake of the progenitors, there was no statistical difference between the groups. The mothers who had the analyzed milk are representatives of their groups when evaluated body weight and feed intake.

The animals from the groups experimental presented anticipations of certain reflexes, as well as improvements in memory. These findings are important because they demonstrate the benefits that CLA consumption can bring to the developing brain. The present research will serve to guarantee a safety indication of the consumption of CLA by pregnant and lactating women by doctors and nutritionists. In addition, the supplement used in this research is a trademark and can be freely purchased by individuals and verify the effects of its consumption is very important. However, it is interesting to conduct research with humans to guarantee the similarity of the results found in the present research with women and infants.

## Conclusion

Based on our results, it may be concluded that maternal supplementation with CLA influenced development of the offspring central nervous system, accelerating reflex maturation (Cliff-avoidance, Vibrissa-placing, Negative-geotaxis, Auditory-startle response) and delay in only two reflexes (Palmar grasp and Free-fall righting) in the group which received 3% CLA. In the group which received 1% CLA was observed acceleration in reflexes (Palmar grasp and Negative-geotaxis). Memory improvement was also observed in CLA treated groups where there was greater exploration of the new object in the object recognition test. It is suggested that further studies be performed to prove the effects of CLA on the central nervous system.

## Ethics Statement

The research followed an experimental protocol in accordance with the ethical recommendations of the National Institutes of Health (Bethesda, MD, United States), and was approved by the ethics research committee of the Federal University of Paraíba No. 0407/13.

## Author Contributions

JS, CB, MM, MO, and RQ designed the theme of the study. MB and ML performed the designed experimental methods. RB, SA, and MS for conducting the analysis of fatty acids. JS, MM, MB, and MQ analyzed the data. JS and MQ interpreted the results and wrote the article. This research was carried out by all authors.

## Conflict of Interest Statement

The authors declare that the research was conducted in the absence of any commercial or financial relationships that could be construed as a potential conflict of interest.
